# Poly-ADP-ribose assisted protein localization resolves that DJ-1, but not LRRK2 or α-synuclein, is localized to the mitochondrial matrix

**DOI:** 10.1371/journal.pone.0219909

**Published:** 2019-07-19

**Authors:** Nelson Osuagwu, Christian Dölle, Charalampos Tzoulis

**Affiliations:** 1 Department of Clinical Medicine, University of Bergen, Bergen, Norway; 2 Department of Neurology, Haukeland University Hospital, Bergen, Norway; 3 Neuro-SysMed Center of Excellence for Clinical Research in Neurological Diseases, Haukeland University Hospital and University of Bergen, Bergen, Norway; University College London, UNITED KINGDOM

## Abstract

Several proteins linked to familial Parkinson disease have been associated with mitochondrial (dys-)function and have been described to reside within mitochondria. The putative mitochondrial and sub-mitochondrial localization of these proteins remains disputed, however, potentially due to conflicting results obtained by diverging technical approaches. Using the high-resolution poly-ADP-ribose assisted protein localization assay that also allows for detection of low level and even partial mitochondrial matrix localization, we demonstrate here that DJ-1, but not LRRK2 or α-synuclein, resides in the mitochondrial matrix. The localization of the proteins was not changed in cellular stress models of Parkinson disease and, in case of α-synuclein, not affected by pathological mutations.

Our results verify the ability of DJ-1 to carry out its role also from within mitochondria and suggest that LRRK2 and α-synuclein may interact with and affect mitochondria from outside the mitochondrial matrix.

## Introduction

Parkinson disease (PD) is a complex disorder influenced by both genetic and environmental factors [1–4]. However, only about 10% of all cases can be linked to genetic causes [5], thus the majority of cases are sporadic with unknown aetiology. While disease mechanisms still remain largely unclear, it is now well established that mitochondrial dysfunction plays a central role in both familial and sporadic PD [6]. This includes, among others, changes in mitochondrial quality control pathways such as mitochondrial DNA homeostasis and mitophagy [7,8], as well as metabolic changes such as complex I deficiency of the mitochondrial respiratory chain [9,10]. Interestingly, several proteins that have been genetically linked to PD are involved in mitochondrial homeostasis and quality control, including PTEN-induced kinase 1 (PINK1), Parkin, DJ-1 and Leucine-rich repeat kinase 2 (LRRK2) [11–13]. PINK1 and Parkin have been described to protect mitochondria against cellular damage and mediate clearance of damaged mitochondria by mitophagy [14]. LRRK2, a protein kinase, is the most commonly mutated protein in familial PD cases [15], while DJ-1 is reported to have neuroprotective function in PD models [16]. α-synuclein, the major component of Lewy bodies found in PD brains, has also been implicated in mitochondrial dysfunction, for example by affecting mitochondrial complex I activity [17].

Several of these proteins have been described to localize partially or entirely to mitochondria, however, in some cases current evidence is conflicting. For example, it is widely established that PINK1 localizes to the mitochondria and is required for Parkin recruitment to the organelles to orchestrate the process of mitophagy [18–20]. DJ-1 has been described to be partially localized to the mitochondria, but its sub-mitochondrial localization remains unclear. Some reports described LRRK2 to be associated with mitochondria [21], while others could not recapitulate these findings [22]. For α-synuclein, few reports suggested mitochondrial localization based on interaction with mitochondrial proteins. Thus, conflicting reports indicate that the mitochondrial localization of DJ-1, LRRK2 and α-synuclein, and particularly the exact sub-mitochondrial localization, which has a direct impact on putative function and interaction, remains to be resolved.

In part, this discrepancy may be due to technical limitations of the most common approaches, such as subcellular fractionation and immunocytochemistry. While subcellular fractionation may present false positive results due to fraction contamination, conventional immunocytochemistry does not distinguish between *intra*-organellar localization and mere association with the organelle from outside.

The recently established *poly-ADP-ribose assisted protein localization assay* (PARAPLAY) resolves this problem of conventional immunocytochemistry and is able to conclusively establish intra-organellar localization [23]. The protein of interest is fused to the catalytic domain of poly-ADP-ribose polymerase 1 (PARP1), termedPARP1cd, which uses NAD^+^ as substrate to generate the immunodetectable biopolymer poly-ADP-ribose (PAR). The subcellular localization of the fusion construct is entirely dependent on the protein of interest. PAR formation is only detectable in the lumen of organelles where sufficient substrate concentration and absence of strong PAR-degrading activity allow for accumulation of PAR [23,24]. Expression of a cytosolic PARP1cd-fusion construct does not lead to detectable PAR formation [23]. Importantly, in mitochondria only proteins that reside in the mitochondrial matrix are able to generate detectable PAR levels, whereas association with the organelle from the outside or intermembrane space localization does not support a robust PAR signal [23]. By combining detection of the recombinant protein itself with the use of PAR formation as readout, intra-organellar (in this study: mitochondrial matrix) localization of the protein is readily established. Importantly, the assay is capable of revealing even partial intra-mitochondrial localization, i.e. when the majority of the protein resides in the cytosol and organellar structures are “hidden” under the cytosolic signal [23].

Here, we investigated three proteins, DJ-1, LRRK2 and α-synuclein, which are all linked to monogenic cases of familial PD, and which have been described to be associated with or located to mitochondria. Using PARAPLAY, we show that DJ-1 is partially localized to the mitochondrial matrix in addition to its cytosolic localization, which remains undiscovered using conventional immunocytochemistry and protein detection alone. In contrast, LRRK2 and α-synuclein do not exhibit intra-mitochondrial localization.

## Materials and methods

### Chemicals, reagents and media

The following antibodies were used: rabbit and mouse (10H) anti poly-ADP-ribose (ALX-210-890A-01900 and ALX-804-220, respectively, Enzo Life Sciences), rabbit and mouse (M2) anti FLAG (F7425 and F3165, respectively, Sigma-Aldrich), mouse anti myc (9E10, TA150121, Origene) and rabbit anti NDUFB10 (ab196019, Abcam). Secondary antibodies goat anti mouse 488 (A11001), goat anti rabbit 594 (A11012) and goat anti rabbit 647 (A21245) were from Life Technologies. DNA-modifying and restriction enzymes were from Thermo Fisher Scientific. All cell culture reagents were from Life Technologies, except Carbonyl-cyanide 3-chlorophenyhydrazone (CCCP) and paraquat (Sigma-Aldrich).

### Generation of eukaryotic expression vectors

The open reading frame (ORF) encoding full-length DJ-1 was amplified from HEK293 cells cDNA, while the ORFs encoding the N-terminal domain of LRRK2 (amino acids 1–266, [25]) and full-length α-synuclein were amplified from pre-existing plasmids (Addgene: pDEST53-LRRK2-WT #25044 and EGFP-alpha synuclein-WT #40822, respectively). All ORFs were inserted into pFLAG-CMV-5a (Sigma-Aldrich) and pcDNA3.1(+)-PARP1cd [23] vectors. ORFs encoding α-synuclein mutants (E46K and A53T) were generated by PCR-based site-directed mutagenesis. All cloned DNA sequences were verified by DNA sequence analysis.

### Cell culture

HeLa S3 cells were cultivated in Ham’s F12 Glutamax nutrient growth medium and SH-SY5Y cells were cultivated in DMEM/Ham`s F12 (1:1) Glutamax medium, both supplemented with 10% (v/v) fetal bovine serum, 100 U/ml penicillin and 100 μg/ml streptomycin and maintained at 37 °C in a humidified atmosphere with 5% CO_2_. Transient transfection was performed using Effectene reagent (Qiagen) according to the manufacturer’s specifications. For pharmacological treatments, 24 h after transfection cells were incubated with 20 μM CCCP for 6 hours or 1 mM (SH-SY5Y) or 2 mM paraquat (HeLa S3) for 24 hours, prior to immunocytochemistry analysis.

### Immunocytochemistry

Cells grown on coverslips were fixed with 3.7% (v/v) paraformaldehyde in PBS at 4 °C for 45 min, followed by permeabilization with 0.5% (v/v) Triton X-100 in PBS for 15 min at room temperature. In some cases, cells were treated with 200 nM Mito Tracker Red CMXRos (Sigma-Aldrich) for 30 min at 37 °C prior to fixation. A blocking step with growth medium containing 10% (v/v) FBS for 1 h at room temperature was followed by overnight incubation with primary antibodies in growth medium at 4 °C. After washing 4 times for 5 min in PBS, the cells were further incubated with secondary antibodies in growth medium for 1 h at room temperature. Cells were washed once for 5 min with PBS and the nuclei were stained with DAPI. After washing twice for 5 min in PBS, the coverslips were mounted with ProLong Diamond Antifade (Invitrogen). Images were acquired using a Leica TCS SP8 confocal laser scanning microscope (Leica Microsystems) with a 100x oil immersion objective (numerical aperture 1.40).

## Results

### PARAPLAY analysis reveals partial localization of DJ-1 to the mitochondrial matrix

**Fig 1:**
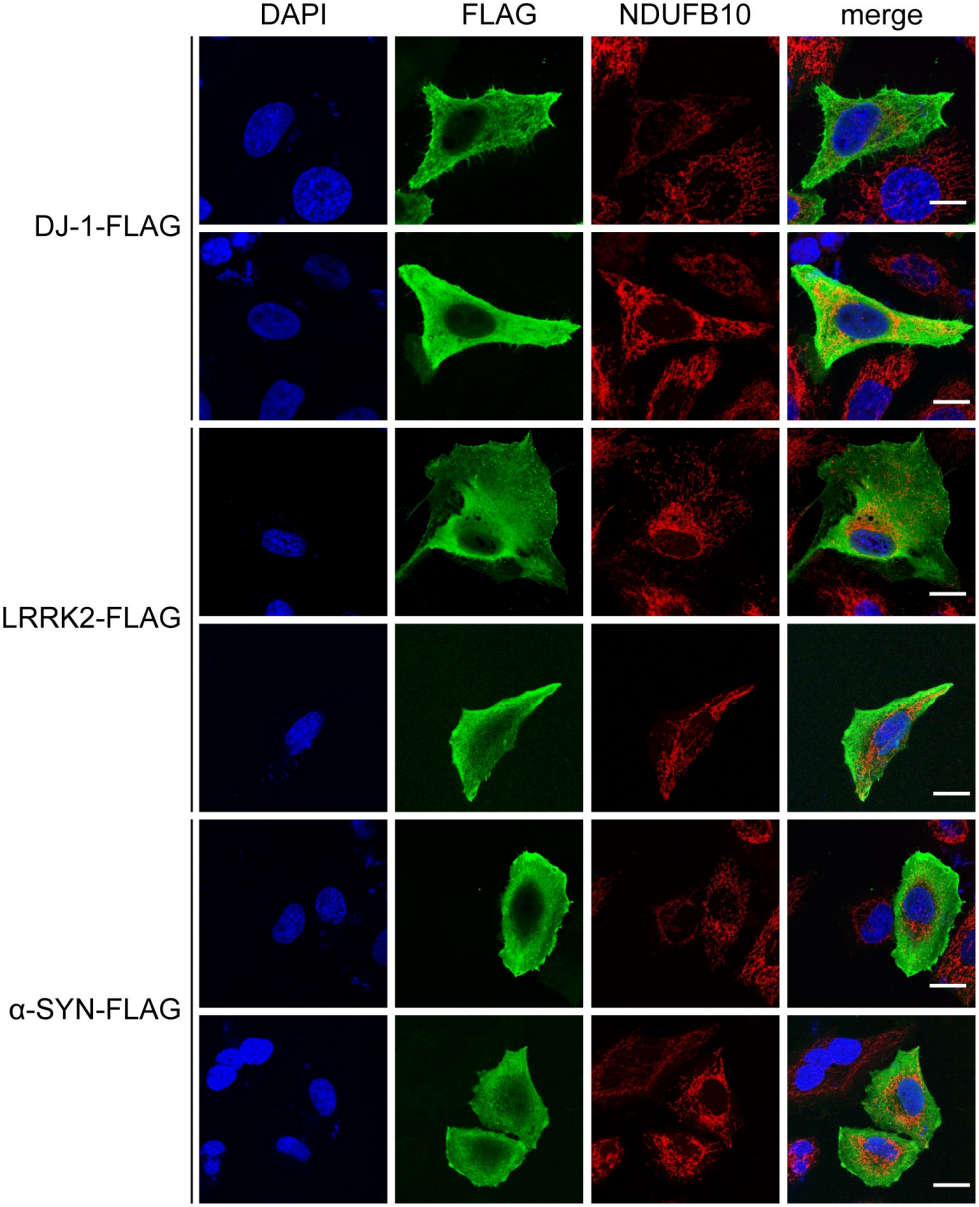
Apparent cytosolic localization of recombinant DJ-1, LRRK2 and α-synuclein. HeLa S3 cells were transiently transfected with constructs encoding C-terminally FLAG-tagged DJ-1, LRRK2 and α-synuclein and subjected to indirect FLAG-immunocytochemistry. Two fluorescence images for each fusion construct are shown, displaying the overexpressed proteins (FLAG), mitochondria (NDUFB10) and the nuclei (DAPI). Scale bar: 10 μm.

In order to investigate the subcellular localization of the proteins of interest in detail, we first overexpressed DJ-1, α-synuclein and the N-terminal domain of LRRK2 (subsequently referred to as “LRRK2”) as FLAG-tagged recombinant proteins which were subsequently detected by conventional immunocytochemistry. DJ-1, LRRK2 and α-synuclein were detected in the cytosol and did not show any colocalization with mitochondrial structures detected by the mitochondrial marker NDUFB10 (Fig 1).

**Fig 2:**
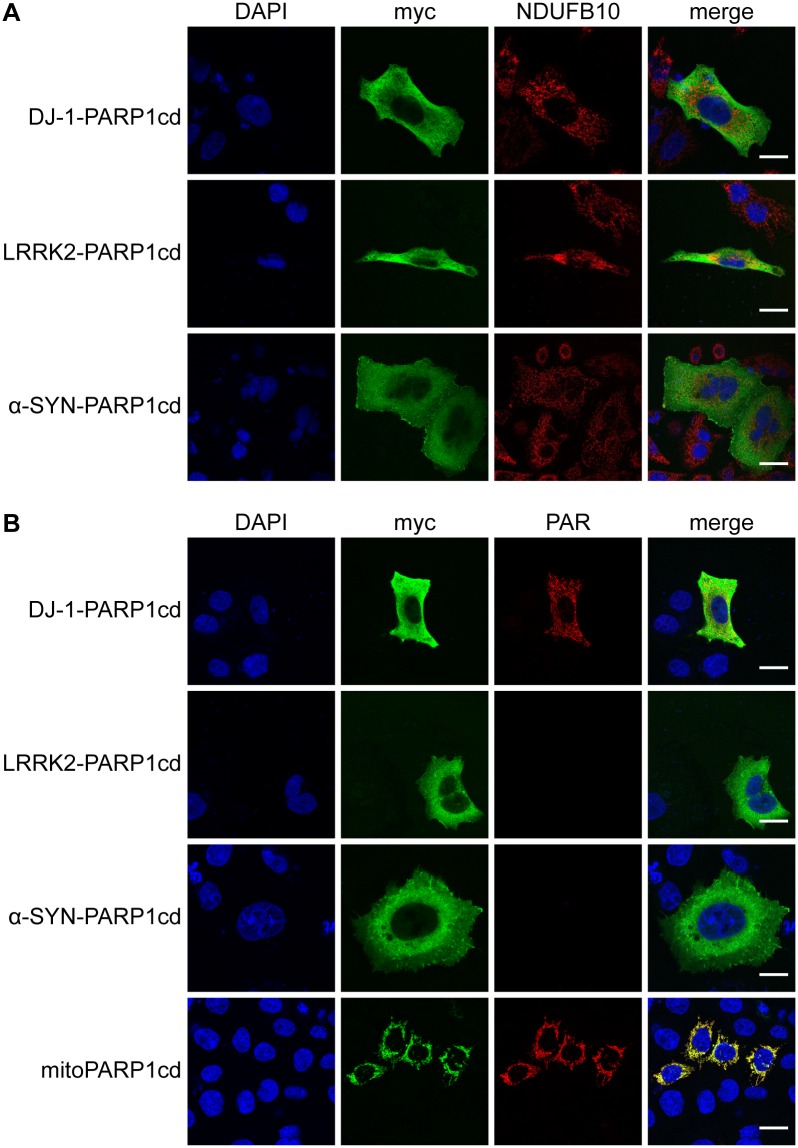
Recombinant DJ-1, but not LRRK2 and α-synuclein, localizes partially to the mitochondrial matrix as revealed by PARAPLAY. HeLa S3 cells were transiently transfected with PARP1cd fusion constructs of DJ-1, LRRK2 and α-synuclein and subjected to indirect immunocytochemistry, detecting the recombinant protein by its myc-epitope and either a mitochondrial marker (A) or PAR accumulation (B). (A) The fluorescent images show the overexpressed proteins (myc), mitochondria (NDUFB10) and the nuclei (DAPI). (B) The fluorescent images show the overexpressed proteins (myc), PAR accumulation (PAR) and the nuclei (DAPI). The mitochondrial matrix-targeted fusion protein mitoPARP1cd served as positive control for intra-mitochondrial PAR formation. Scale bar: 10 μm.

Next, we performed PAR-assisted protein localization assay, PARAPLAY [23], using constructs of DJ-1, LRRK2 and α-synuclein C-terminally fused to PARP1cd and a myc-epitope for protein detection. Again, when overexpressed in cells, DJ-1, α-synuclein and LRRK2 fusion proteins were detected in the cytosol and did not show colocalization with mitochondria (Fig 2A, S1A Fig). This confirmed that fusion to PAPR1cd did not change subcellular localization.

Importantly, when cells were subjected to PAR immunocytochemistry, cells overexpressing the DJ-1-PARP1cd fusion protein showed a positive PAR signal that did not colocalize with the diffuse, cytosolic pattern of the detected protein (Fig 2B, S1B Fig), but in fact colocalized with mitochondria (S2 Fig). Using a mitochondrial matrix-targeted EGFP-PARP1cd-fusion protein, termed mitoPARP1cd [24], we confirmed the specificity of the PAR signal (Fig 2B, S1B Fig). In contrast, PAR formation was not observed for the fusion constructs of α-synuclein or LRRK2 (Fig 2B, S1B Fig). We repeated this experiment using the neuroblastoma SH-SY5Y cell line and confirmed that only DJ-1-PARP1cd expression led to detectable polymer formation, while expression of all other constructs did not (S3 Fig). These results indicated that a portion of DJ-1 localized to the mitochondrial matrix, while α-synuclein and LRRK2 did not.

### Subcellular localization is unchanged in toxin-induced models of PD

**Fig 3:**
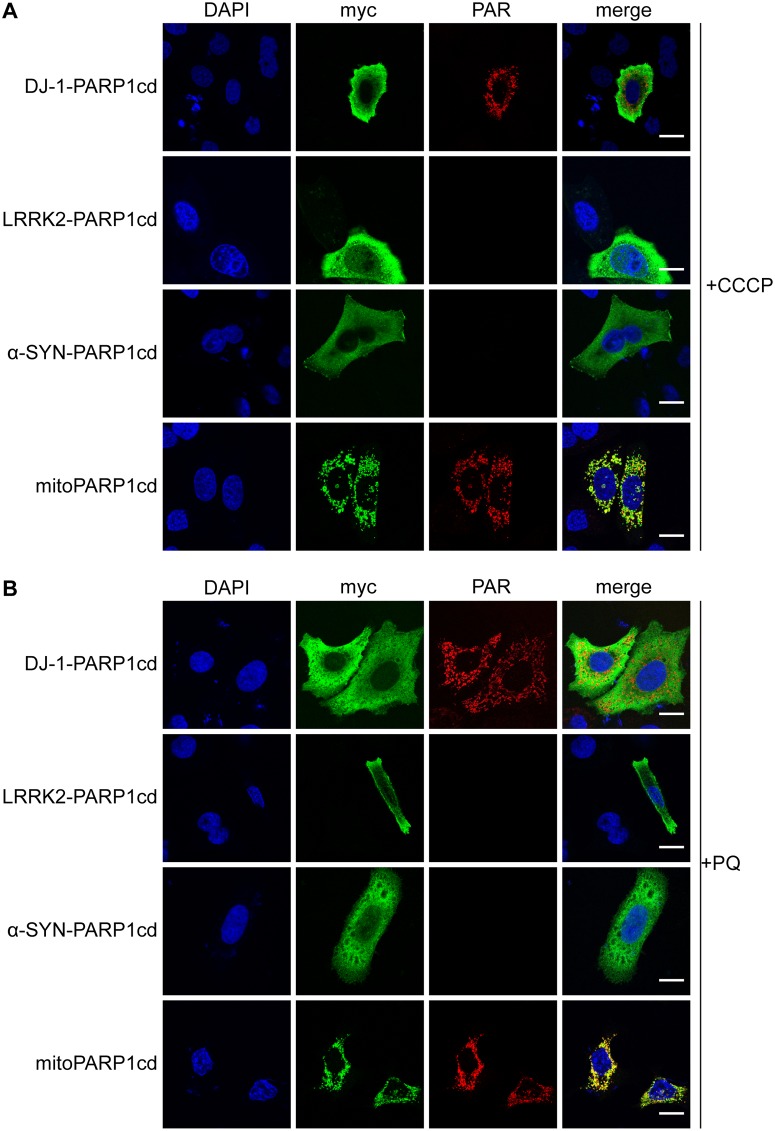
CCCP and paraquat treatment does not affect subcellular localization of recombinant DJ-1, LRRK2 and α-synuclein. Transiently transfected HeLa S3 cells were treated 24 hours after transfection with 20 μM CCCP for 6 hours (A) or 2 mM paraquat (PQ) for 24 hours (B) and subjected to myc and PAR immunocytochemistry. The fluorescent images show overexpressed proteins (myc), PAR accumulation (PAR) and the nuclei (DAPI). MitoPARP1cd served as positive control. Scale bar: 10 μm.

Previous studies have linked mitochondrial dysfunction and loss of membrane potential to PD. In some cases, these changes of mitochondrial membrane potential could affect protein localization and lead to protein accumulation within mitochondria [18,26]. We therefore tested whether the loss of mitochondrial membrane potential could influence the localization of the proteins of interest in our system. Upon treatment with the ionophore CCCP, the mitochondrial membrane potential collapsed (S4 Fig). Overexpression of mitoPARP1cd still resulted in PAR accumulation, indicating that the treatment did not interfere with the detection system (Fig 3A, S5A Fig). More importantly, while PAR formation was still detected for DJ-1-PARP1cd (Fig 3A, S5A Fig), there was no detectable increase in PAR formation or change in the detection of the protein in the cytosol (S6 Fig). This indicated that the partial mitochondrial matrix localization of DJ-1 protein was not affected and likely not to be dependent on the mitochondrial membrane potential. LRRK2 and α-synuclein still did not reveal any detectable PAR formation, suggesting that also their subcellular localization was unaltered and these proteins were not localized to the mitochondrial matrix under stress conditions (Fig 3A, S5A and S6 Figs).

Paraquat is a chemical toxin that causes a PD-like phenotype in rodents [27] and affects mitochondrial function, leading to production of reactive oxygen species and cell death [28]. As representative for parkinsonism-inducing agents, we next investigated the effect of paraquat on the subcellular localization of these proteins. Pharmacological treatment with paraquat for 24 h did not lead to increased association of the overexpressed proteins with mitochondria (S7 Fig). Moreover, the PAR signal resulting from DJ-1-PARP1cd overexpression was still present, but not increased, while no PAR formation was detectable in case of LRRK2 and α-synuclein (Fig 3B, S5B Fig). Prolonged incubation with paraquat for up to 48 h did not change protein localization or led to detectable PAR formation.

In order to exclude cell type-specific effects we reproduced the experiments under cellular stress conditions in SH-SY5Y cells. Importantly, neither CCCP nor paraquat treatment changed the detectable PAR formation for any of the fusion constructs also in these cells (S8 Fig).

### α-synuclein mutations A53T and E46K do not mediate mitochondrial matrix localization

It has been described that pathogenic α-synuclein mutations are associated with mitochondrial dysfunction, such as increased degree of mitochondrial fragmentation [29]. We hypothesized that these effects may be partly mediated by mitochondrial relocation of the mutant α-synuclein protein. To test this hypothesis, we investigated whether the common α-synuclein mutations A53T and E46K lead to the relocation of the protein to the mitochondrial matrix.

**Fig 4:**
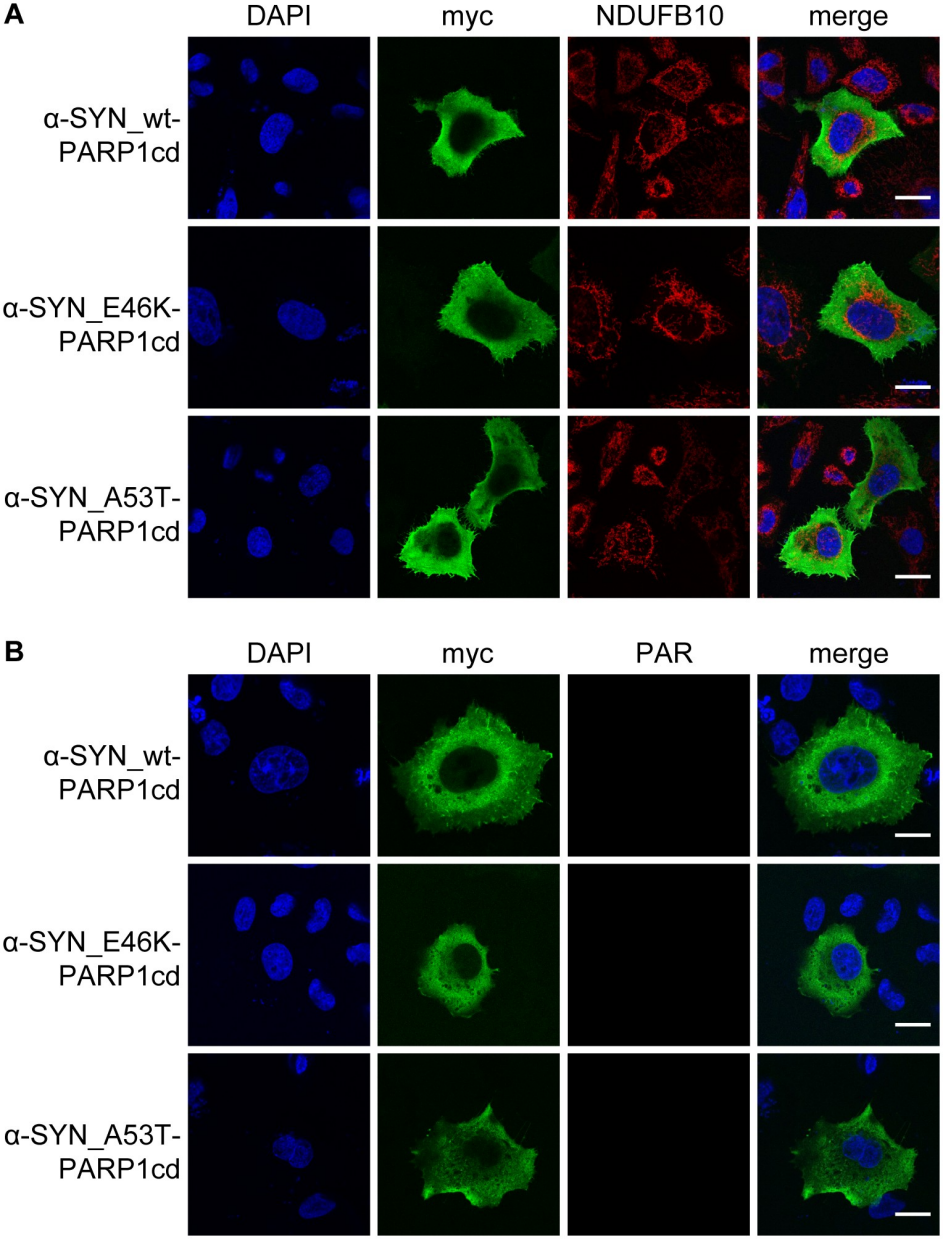
α-synuclein mutant forms are absent from mitochondrial matrix. HeLa S3 cells were transiently transfected with PARP1cd fusion constructs of α-synuclein wild type (wt) and mutants (E46K and A53T) and subsequently subjected to indirect immunocytochemistry detecting the recombinant protein by its myc epitope and either a mitochondrial marker (A) or PAR accumulation (B). (A) The fluorescent images show the overexpressed proteins (myc), mitochondria (NDUFB10) and nuclei (DAPI). (B) The fluorescent images show the overexpressed proteins (myc), PAR accumulation (PAR) and the nuclei (DAPI). Scale bar: 10 μm.

PARAPLAY analysis revealed the absence of the recombinant proteins from mitochondria (Fig 4). An apparent cytosolic localization was detected for the PARP1cd fusion constructs (Fig 4A, S1A Fig) and PAR formation was not observed for either of the mutated proteins in both HeLa S3 (Fig 4B, S1B Fig) and SH-SY5Y cells (S3 Fig), similar to the wild type protein. Furthermore, cytotoxic stress by loss of mitochondrial membrane potential (S9 Fig) or paraquat treatment (S10 Fig) did not change the subcellular localization of the mutated proteins towards the intra-mitochondrial compartment in both HeLa S3 (S9 and S10 Figs) and SH-S5Y5 cells (S11 Fig).

## Discussion

The involvement of mitochondrial dysfunction in PD is widely accepted, yet its exact role and contribution to the progression of the disease remains elusive. The determination of the exact subcellular localizations of PD-linked proteins is one major step to elucidate their contribution to mitochondrial dysfunction and to reveal underlying mechanisms. We report here that DJ-1 is present in the mitochondrial matrix under normal and stress conditions in human cells, while LRRK2 and α-synuclein are absent from the mitochondrial matrix.

The subcellular distribution of DJ-1 has previously been addressed in several studies and it has been reported to localize to the nucleus, the cytosol and the mitochondria. However, especially with regard to its putative sub-mitochondrial distribution, varying and partially conflicting results were reported. Previous studies showed, among others, an association with the mitochondrial outer membrane [30] and that DJ-1 localizes to the mitochondria only in some circumstances or relocates to mitochondria upon cellular stress [31]. Others stated that only mutant DJ-1, but not wild type, localizes to the mitochondrial matrix [32]. Our demonstration that wild type DJ-1 is already present in the mitochondrial matrix under normal conditions further corroborates its important role for mitochondria, enabling it to carry out its cytoprotective function directly at the site of putative oxidative damage and to immediately react to potential insults. Moreover, the loss-of-function mutations found in PD are thus more prone to affect mitochondrial function directly.

In contrast to DJ-1, LRRK2 did not localize to the mitochondrial matrix under any conditions tested in this study. Previously, localization to membranous and vesicular structures in the human brain [33] and a partial association with the outer mitochondrial membrane [21] have been reported for LRRK2, while other studies found LRRK2 mainly in the cytosol and could not recapitulate mitochondrial association [22]. We also detected the recombinant LRRK2 protein rather distributed throughout the cytosol. However, as our results for DJ-1 clearly showed, apparent cytosolic distribution may conceal association with organelles and therefore, we cannot rule out any association with mitochondria or other organelles from the outside. Moreover, we cannot completely rule out the possibility that the full length LRRK2 protein still may localize to mitochondria, although it is in the N-terminal domain where a putative mitochondrial targeting sequence is most often located. However, the absence of our fusion protein from the mitochondrial matrix demonstrated by the lack of PAR signal is consistent with the proposed role of LRRK2 in mitochondrial dynamics, for example by functional interaction with proteins regulating mitochondrial fission (Drp1) and fusion (mitofusins and OPA1) [34].

The endogenous function of α-synuclein has yet to be fully elucidated and defining its subcellular distribution is of great importance in this regard. While it was originally described to localize to the nucleus and the cytosol, more recent findings indicated also localization to mitochondria [35] and mitochondria-associated ER membranes [29]. Especially its described interaction with complex I of the mitochondrial respiratory chain and ATP synthase, both localized to the inner mitochondrial membrane, raised the question whether this interaction results from binding from the outside or inside of the organelle.

Our results strongly suggest that α-synuclein is absent from the mitochondrial matrix, both under normal and stress conditions. Moreover, PD-causing mutations were also not localized to the mitochondrial matrix. This does not contradict the putative role of α-synuclein in mitochondrial dysfunction and interaction with complexes of the mitochondrial respiratory chain, but rather reveals that possible interaction would necessarily take place on the intermembrane space facing part of these complexes. This possibility is further supported by the recent report of protein interaction between α-synuclein and TOM20, a transport protein localized in the outer mitochondrial membrane [36].

## Conclusion

Our study confirms that the exact determination of subcellular protein distribution in general is challenging and that conclusions need to be drawn with care, based on reliable and well-controlled results. This is particularly true for mitochondria, which contain multiple closely connected sub-organellar compartments. On the other hand, while we here report the *lack* of intra-mitochondrial localization for two of the investigated proteins, our results also suggest that there may be (in fact, many) more proteins *partially* localized to mitochondria and especially the mitochondrial matrix, which would open a whole new spectrum of interaction partners, substrates and functional impact for those candidates.

We therefore suggest that reported intra-mitochondrial protein localization, or lack thereof, based on conventional immunodetection is evaluated with care, as partial intra-organellar localization is easily missed. We recommend the straight-forward PARAPLAY approach as additional detection system in case of suspected organellar association and impact of proteins of interest on organellar, especially mitochondrial function.

## Supporting information

S1 Fig Recombinant DJ-1, but not LRRK2 and wildtype or mutant α-synuclein, localizes partially to the mitochondrial matrix as revealed by PARAPLAY in HeLa S3 cells. Additional images of HeLa S3 cells transiently transfected with PARP1cd fusion constructs of DJ-1, LRRK2 and α-synuclein wild-type or PD-relevant mutants and subjected to indirect immunocytochemistry, detecting the recombinant protein by its myc-epitope and either a mitochondrial marker (A) or PAR accumulation (B) are shown. (A) The fluorescent images show the overexpressed proteins (myc), mitochondria (NDUFB10) and the nuclei (DAPI). (B) The fluorescent images show the overexpressed proteins (myc), PAR accumulation (PAR) and the nuclei (DAPI). The mitochondrial matrix-targeted fusion protein mitoPARP1cd served as positive control for intra-mitochondrial PAR formation. Scale bar: 10 μm.
(TIF)

S2 Fig DJ-1-PARP1cd dependent PAR formation localizes to mitochondria. HeLa S3 cells were transiently transfected with DJ-1-PARP1cd fusion construct and subjected to immunocytochemical analysis. The fluorescent images show the overexpressed proteins (myc), PAR accumulation (PAR), mitochondria (NDUFB10) and nuclei (DAPI). Scale bar: 10 μm.
(TIF)

S3 Fig Recombinant DJ-1, but not LRRK2 and wildtype or mutant α-synuclein, localizes partially to the mitochondrial matrix in SH-SY5Y cells. Neuroblastoma SH-SY5Y cells were transiently transfected with PARP1cd-fusion constructs of DJ1, LRRK2, α -synuclein wild type and PD-relevant α-synuclein mutants and subjected to indirect immunocytochemistry detecting the recombinant protein by its myc-epitope and PAR accumulation. The fluorescent images show the overexpressed proteins (myc), PAR accumulation (PAR) and the nuclei (DAPI). The mitochondrial matrix-targeted fusion protein mitoPARP1cd served as positive control for intra-mitochondrial PAR formation. Scale bar: 10 μm.
(TIF)

S4 Fig Loss of mitochondrial membrane potential upon CCCP treatment. HeLa S3 cells were stained with membrane potential dependent MitoTracker Red CMXRos (MT) after incubation in absence or presence of 20 μM CCCP. DAPI staining of nuclei is shown in blue. Scale bar: 10 μm.
(TIF)

S5 Fig CCCP and paraquat treatment does not affect subcellular localization of recombinant DJ-1, LRRK2 and α-synuclein in HeLa S3 cells. Additional images of transiently transfected HeLa S3 cells treated 24 hours after transfection with 20 μM CCCP for 6 hours (A) or 2 mM paraquat (PQ) for 24 hours (B) and subjected to myc and PAR immunocytochemistry are shown. The fluorescent images show overexpressed proteins (myc), PAR accumulation (PAR) and the nuclei (DAPI). MitoPARP1cd served as positive control. Scale bar: 10 μm.
(TIF)

S6 Fig Loss of mitochondrial membrane potential does not affect subcellular localization of recombinant DJ-1, LRRK2 and α-synuclein. HeLa S3 cells were transiently transfected with either FLAG-tagged (A) or PARP1cd (B) fusion constructs of DJ-1, LRRK2 and α-synuclein and treated 24 hours after transfection with 20 μM CCCP for 6 hours followed by indirect immunocytochemistry. The fluorescent images show the overexpressed proteins (FLAG (A) or myc (B)), mitochondria (NDUFB10) and the nuclei (DAPI). Scale bar: 10 μm.
(TIF)

S7 Fig Paraquat treatment does not affect subcellular localization of recombinant DJ-1, LRRK2 and α-synuclein. HeLa S3 cells transiently transfected with either FLAG-tagged (A) or PARP1cd (B) fusion constructs of DJ-1, LRRK2 and α-synuclein were treated 24 hours after transfection with 2 mM paraquat for 24 hours followed by indirect immunocytochemistry. The fluorescent images show the overexpressed proteins (FLAG (A) or myc (B)), mitochondria (NDUFB10) and the nuclei (DAPI). Scale bar: 10 μm.
(TIF)

S8 Fig CCCP and paraquat treatment does not alter subcellular localization of DJ-1, LRRK2 and α -synuclein in SH-SY5Y cells. SH-S5Y5 cells, transiently transfected with PARP1cd fusion constructs of DJ-1, LRRK2 or α-synuclein, were treated 24 hours after transfection with 20 μM CCCP for 6 hours (A) or 1 mM paraquat for 24 hours (B) and subsequently subjected to indirect immunocytochemistry, detecting the recombinant protein and PAR accumulation (B). The fluorescent images show the overexpressed proteins (myc), PAR accumulation (PAR) and nuclei (DAPI). Scale bar: 10 μm.
(TIF)

S9 Fig Loss of mitochondrial membrane potential does not affect subcellular localization of α-synuclein mutants. HeLa S3 cells, transiently transfected with PARP1cd fusion constructs of α-synuclein wt and mutants (E46K and A53T), were treated 24 hours after transfection with 20 μM CCCP for 6 hours and subsequently subjected to indirect immunocytochemistry, detecting the recombinant protein by its myc epitope and either a mitochondrial marker (A) or PAR accumulation (B). A) The fluorescent images show the overexpressed proteins (myc), mitochondria (NDUFB10) and nuclei (DAPI). Scale bar: 10 μm. (B) The fluorescent images show the overexpressed proteins (myc), PAR accumulation (PAR) and the nuclei (DAPI). Scale bar: 10 μm.
(TIF)

S10 Fig Paraquat treatment does not affect subcellular localization of α-synuclein mutants. HeLa S3 cells, transiently transfected with PARP1cd fusion constructs of α-synuclein wt and mutants (E46K and A53T), were treated 24 hours after transfection with 2 mM paraquat for 24 hours and subsequently subjected to indirect immunocytochemistry detecting the recombinant protein by its myc epitope and either a mitochondrial marker (A) or PAR accumulation (B). (A) The fluorescent images show the overexpressed proteins (myc), mitochondria (NDUFB10) and nuclei (DAPI). Scale bar: 10 μm. (B) The fluorescent images show the overexpressed proteins (myc), PAR accumulation (PAR) and the nuclei (DAPI). Scale bar: 10 μm.
(TIF)

S11 Fig CCCP and paraquat treatment does not affect subcellular localization of α-synuclein mutants in SH-SY5Y cells. SH-SY5Y cells transiently transfected with PARP1cd-fusion constructs of α-synuclein wt and mutants (E46K and A53T), were treated 24 hours after transfection with 20 μM CCCP for 6 hours (A) or 1 mM paraquat (PQ) for 24 hours (B) and subsequently subjected to indirect immunocytochemistry detecting the recombinant protein and PAR accumulation. The fluorescent images show the overexpressed proteins (myc), PAR accumulation (PAR) and nuclei (DAPI). Scale bar: 10 μm.
(TIF)
