# Supplementary figures and images for: Poly-ADP-ribose assisted protein localization resolves that DJ-1, but not LRRK2 or α-synuclein, is localized to the mitochondrial matrix
Source: PLoS One. 2019 Jul 19;14(7):e0219909. doi: 10.1371/journal.pone.0219909 (PMC6641658; doi:10.1371/journal.pone.0219909)

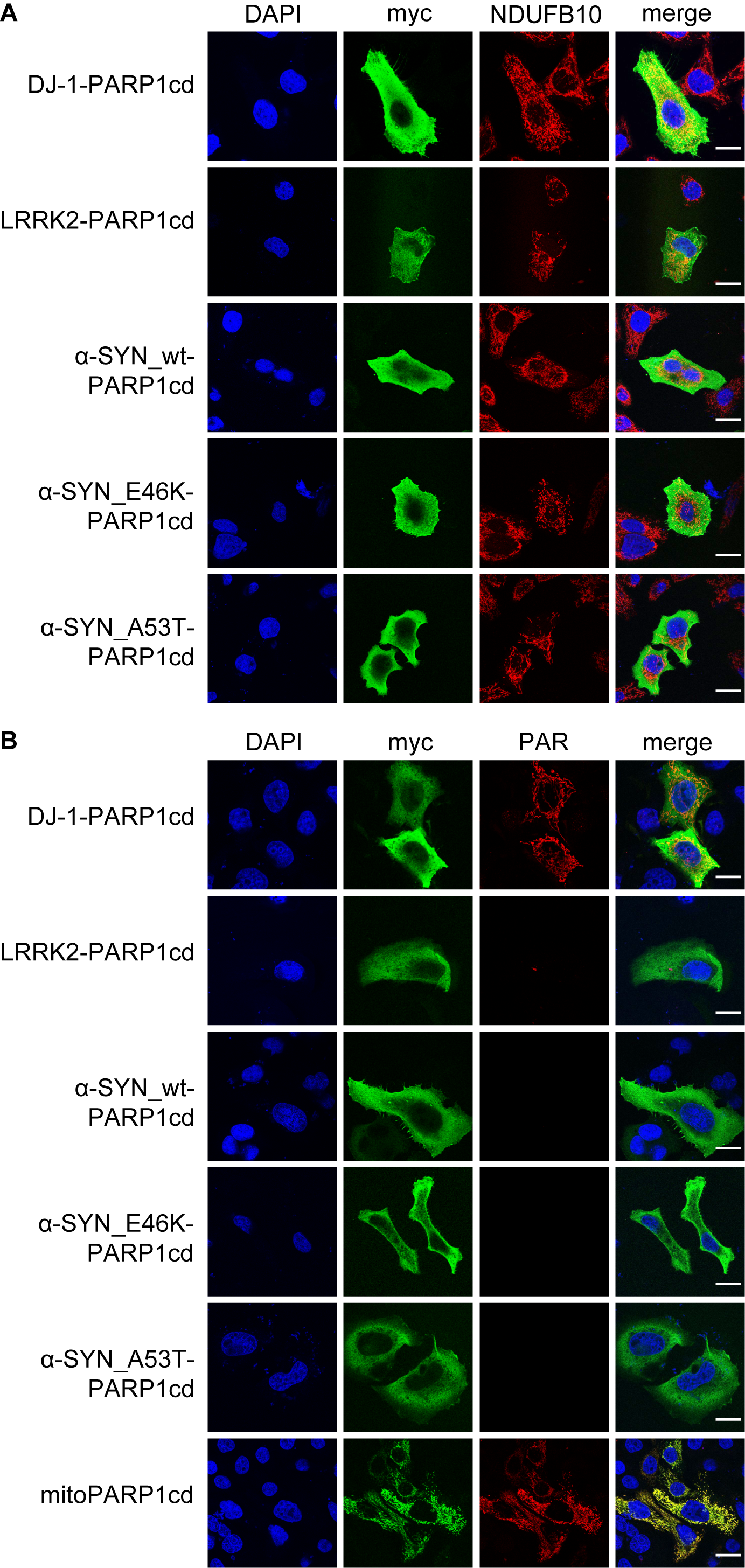

Supplement: S1 Fig — Additional images of HeLa S3 cells transiently transfected with PARP1cd fusion constructs of DJ-1, LRRK2 and α-synuclein wild-type or PD-relevant mutants and subjected to indirect immunocytochemistry, detecting the recombinant protein by its myc-epitope and either a mitochondrial marker (A) or PAR accumulation (B) are shown. (A) The fluorescent images show the overexpressed proteins (myc), mitochondria (NDUFB10) and the nuclei (DAPI). (B) The fluorescent images show the overexpressed proteins (myc), PAR accumulation (PAR) and the nuclei (DAPI). The mitochondrial matrix-targeted fusion protein mitoPARP1cd served as positive control for intra-mitochondrial PAR formation. Scale bar: 10 μm. (TIF) [file pone.0219909.s001.tif]

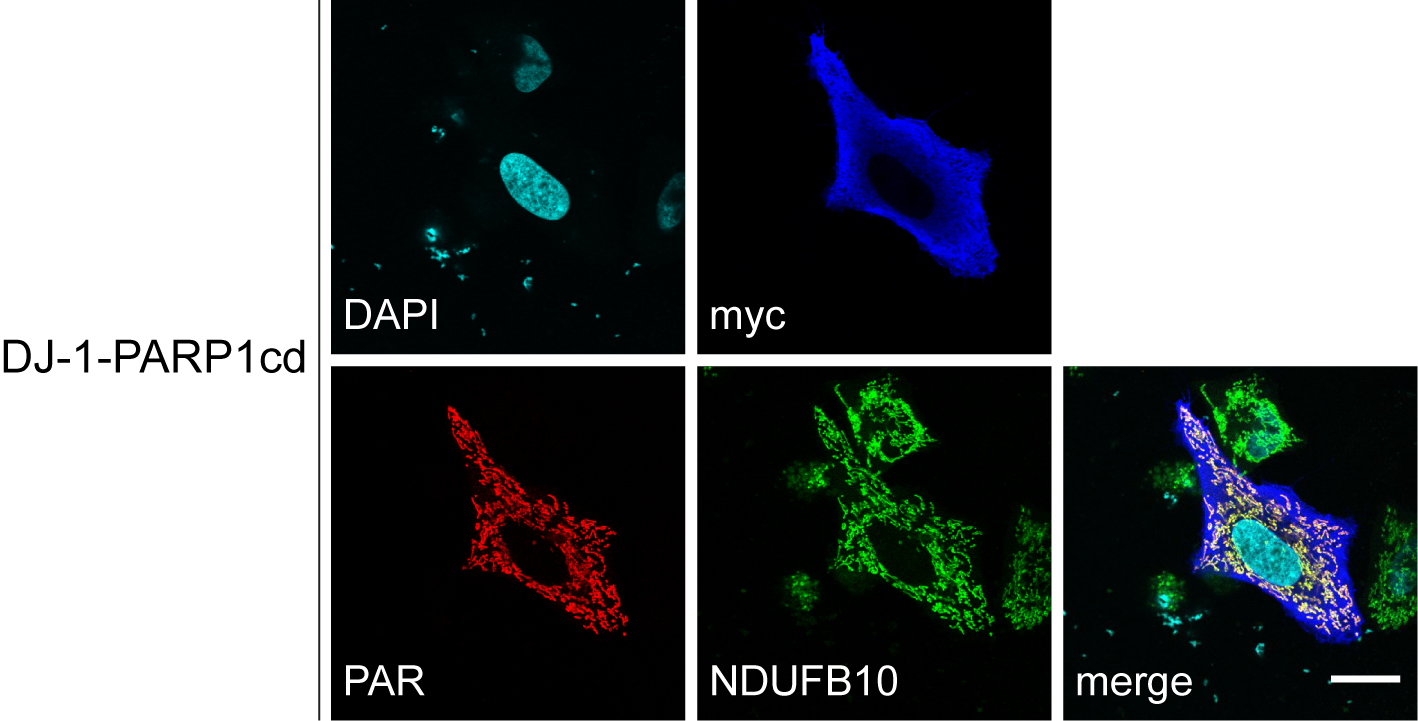

Supplement: S2 Fig — HeLa S3 cells were transiently transfected with DJ-1-PARP1cd fusion construct and subjected to immunocytochemical analysis. The fluorescent images show the overexpressed proteins (myc), PAR accumulation (PAR), mitochondria (NDUFB10) and nuclei (DAPI). Scale bar: 10 μm. (TIF) [file pone.0219909.s002.tif]

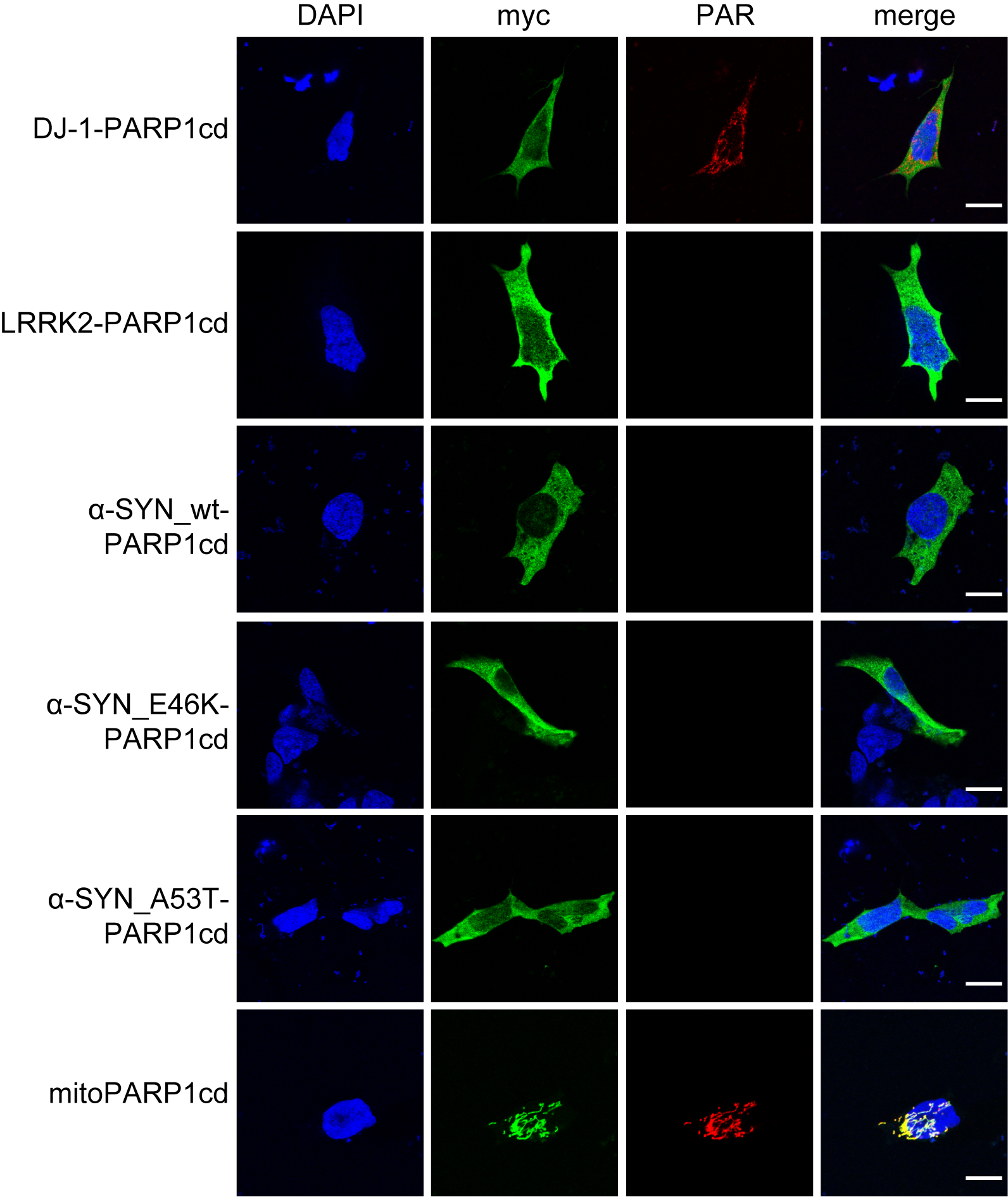

Supplement: S3 Fig — Neuroblastoma SH-SY5Y cells were transiently transfected with PARP1cd-fusion constructs of DJ1, LRRK2, α -synuclein wild type and PD-relevant α-synuclein mutants and subjected to indirect immunocytochemistry detecting the recombinant protein by its myc-epitope and PAR accumulation. The fluorescent images show the overexpressed proteins (myc), PAR accumulation (PAR) and the nuclei (DAPI). The mitochondrial matrix-targeted fusion protein mitoPARP1cd served as positive control for intra-mitochondrial PAR formation. Scale bar: 10 μm. (TIF) [file pone.0219909.s003.tif]

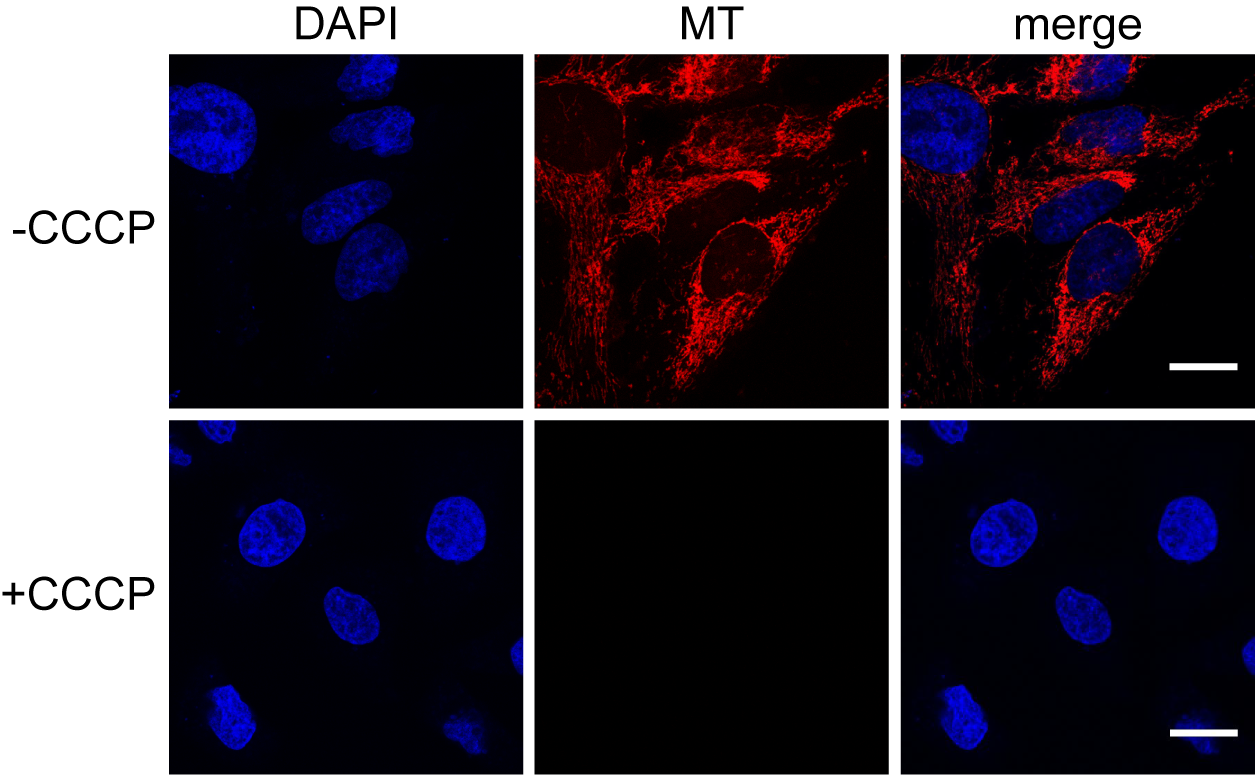

Supplement: S4 Fig — HeLa S3 cells were stained with membrane potential dependent MitoTracker Red CMXRos (MT) after incubation in absence or presence of 20 μM CCCP. DAPI staining of nuclei is shown in blue. Scale bar: 10 μm. (TIF) [file pone.0219909.s004.tif]

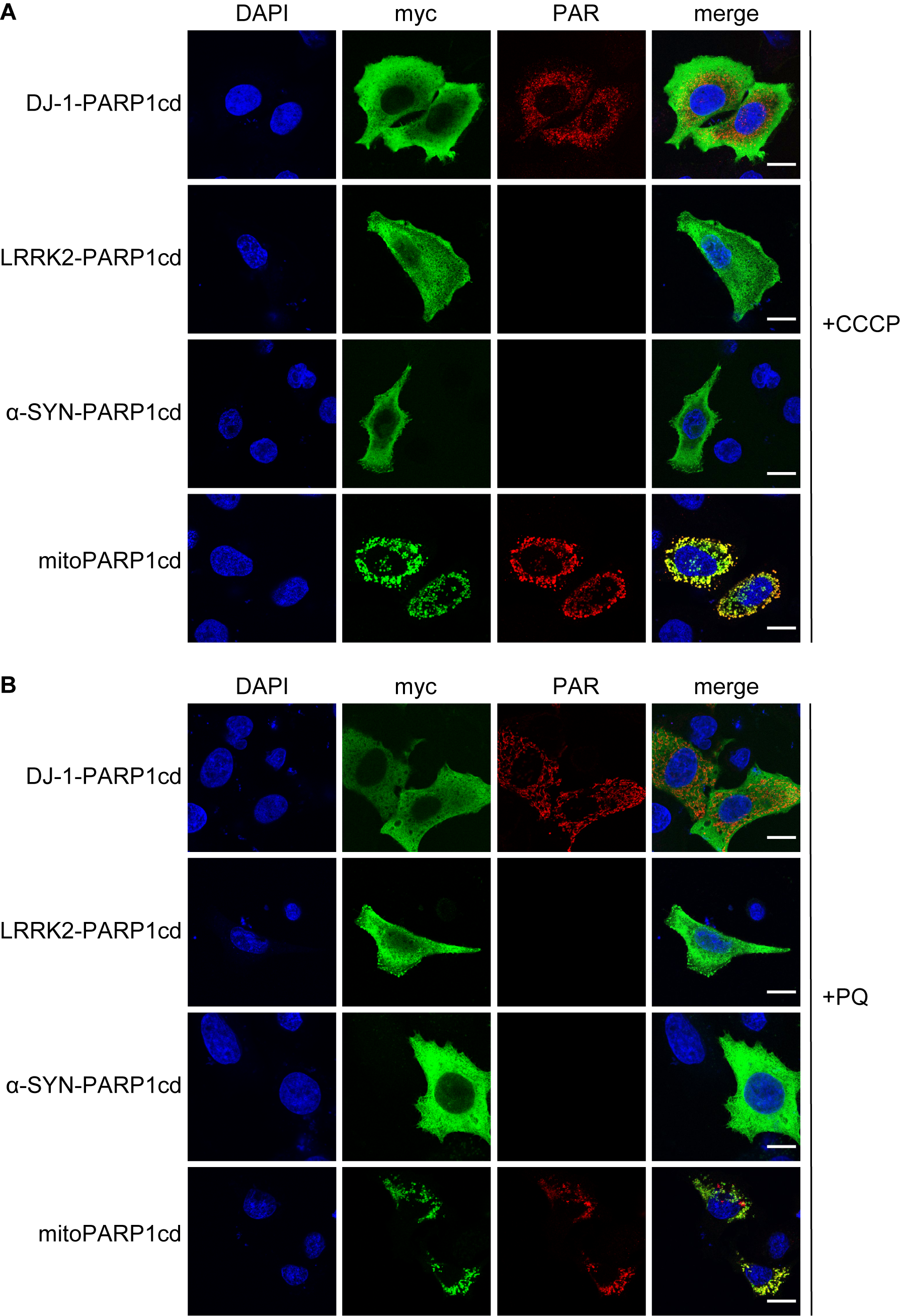

Supplement: S5 Fig — Additional images of transiently transfected HeLa S3 cells treated 24 hours after transfection with 20 μM CCCP for 6 hours (A) or 2 mM paraquat (PQ) for 24 hours (B) and subjected to myc and PAR immunocytochemistry are shown. The fluorescent images show overexpressed proteins (myc), PAR accumulation (PAR) and the nuclei (DAPI). MitoPARP1cd served as positive control. Scale bar: 10 μm. (TIF) [file pone.0219909.s005.tif]

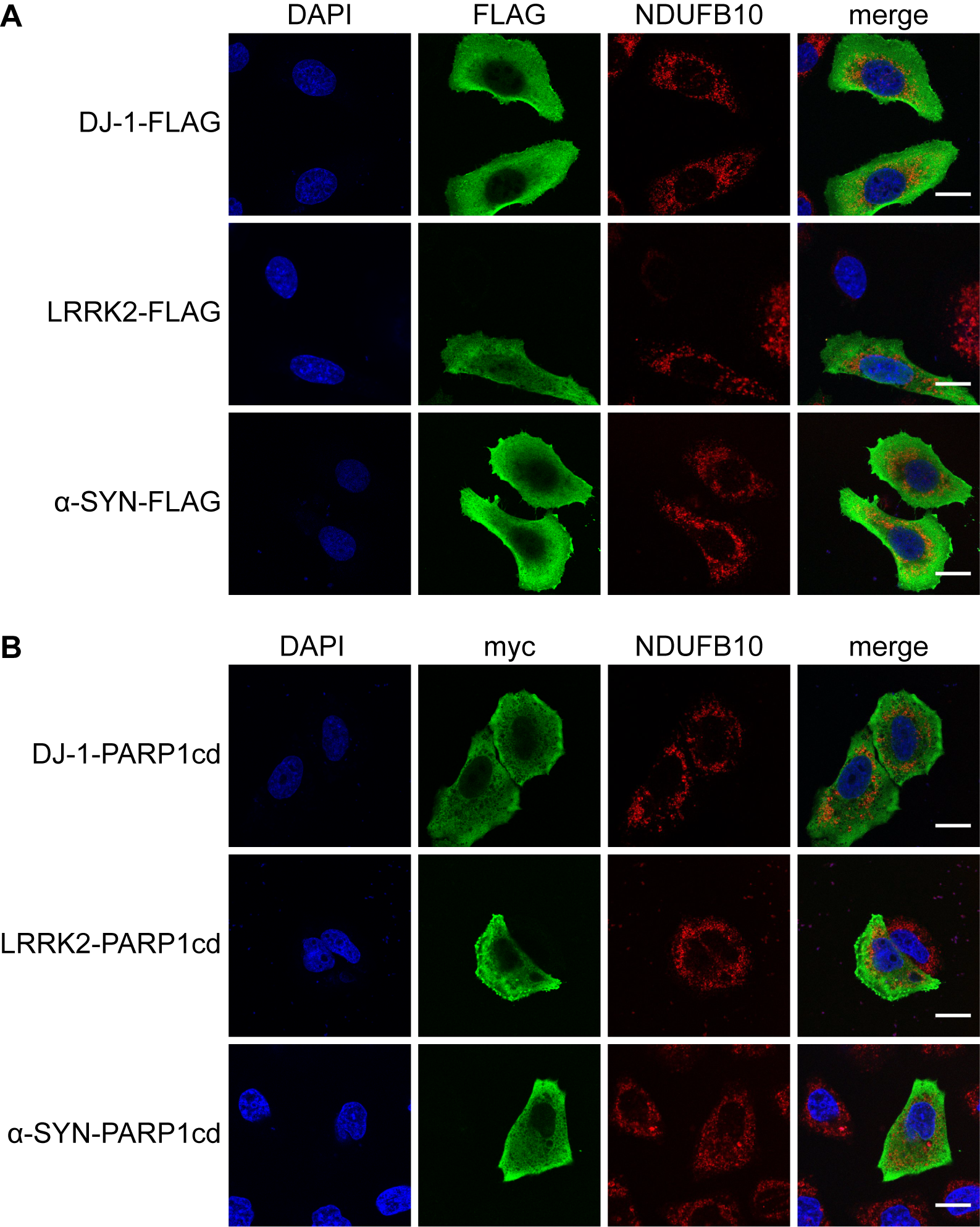

Supplement: S6 Fig — HeLa S3 cells were transiently transfected with either FLAG-tagged (A) or PARP1cd (B) fusion constructs of DJ-1, LRRK2 and α-synuclein and treated 24 hours after transfection with 20 μM CCCP for 6 hours followed by indirect immunocytochemistry. The fluorescent images show the overexpressed proteins (FLAG (A) or myc (B)), mitochondria (NDUFB10) and the nuclei (DAPI). Scale bar: 10 μm. (TIF) [file pone.0219909.s006.tif]

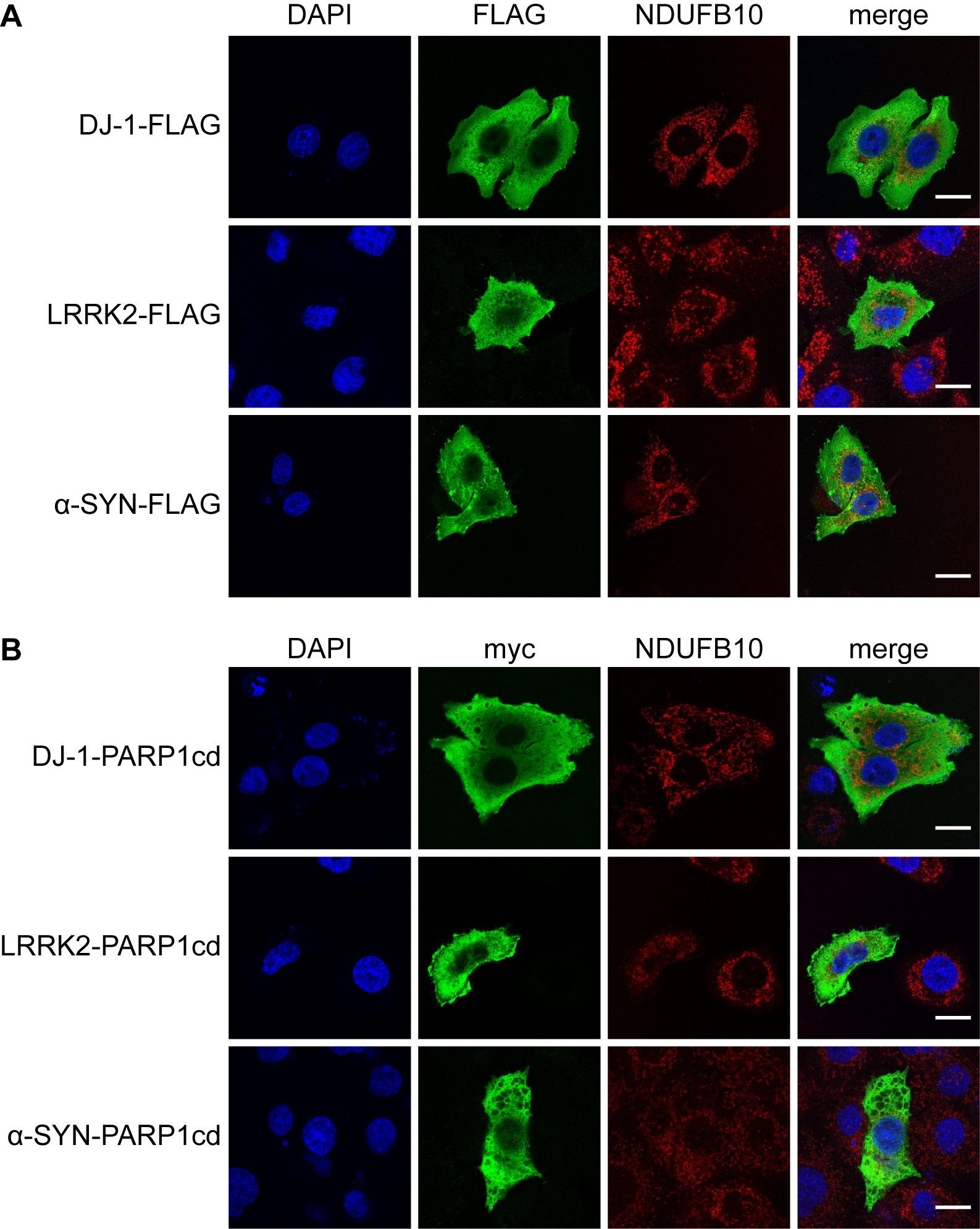

Supplement: S7 Fig — HeLa S3 cells transiently transfected with either FLAG-tagged (A) or PARP1cd (B) fusion constructs of DJ-1, LRRK2 and α-synuclein were treated 24 hours after transfection with 2 mM paraquat for 24 hours followed by indirect immunocytochemistry. The fluorescent images show the overexpressed proteins (FLAG (A) or myc (B)), mitochondria (NDUFB10) and the nuclei (DAPI). Scale bar: 10 μm. (TIF) [file pone.0219909.s007.tif]

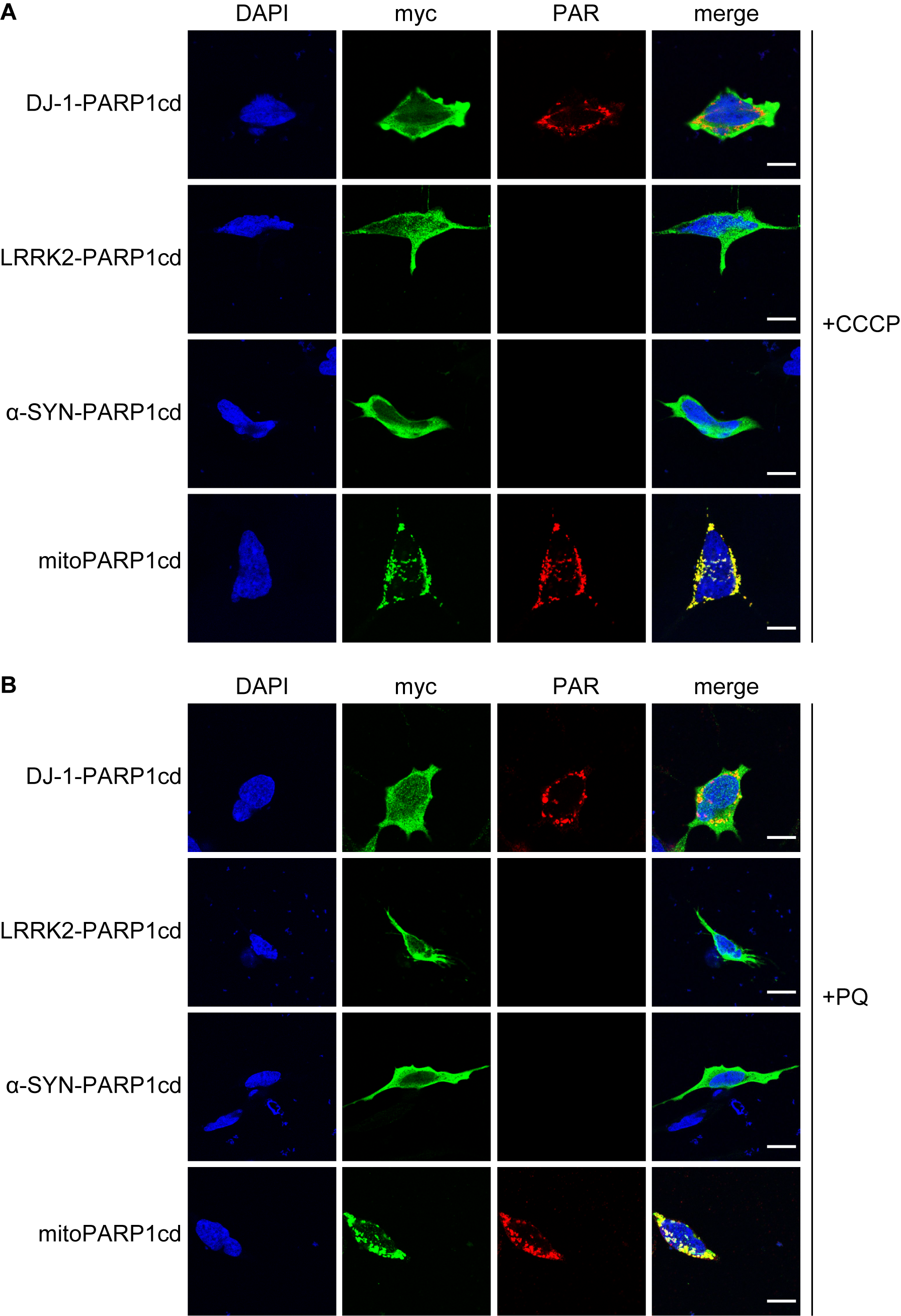

Supplement: S8 Fig — SH-S5Y5 cells, transiently transfected with PARP1cd fusion constructs of DJ-1, LRRK2 or α-synuclein, were treated 24 hours after transfection with 20 μM CCCP for 6 hours (A) or 1 mM paraquat for 24 hours (B) and subsequently subjected to indirect immunocytochemistry, detecting the recombinant protein and PAR accumulation (B). The fluorescent images show the overexpressed proteins (myc), PAR accumulation (PAR) and nuclei (DAPI). Scale bar: 10 μm. (TIF) [file pone.0219909.s008.tif]

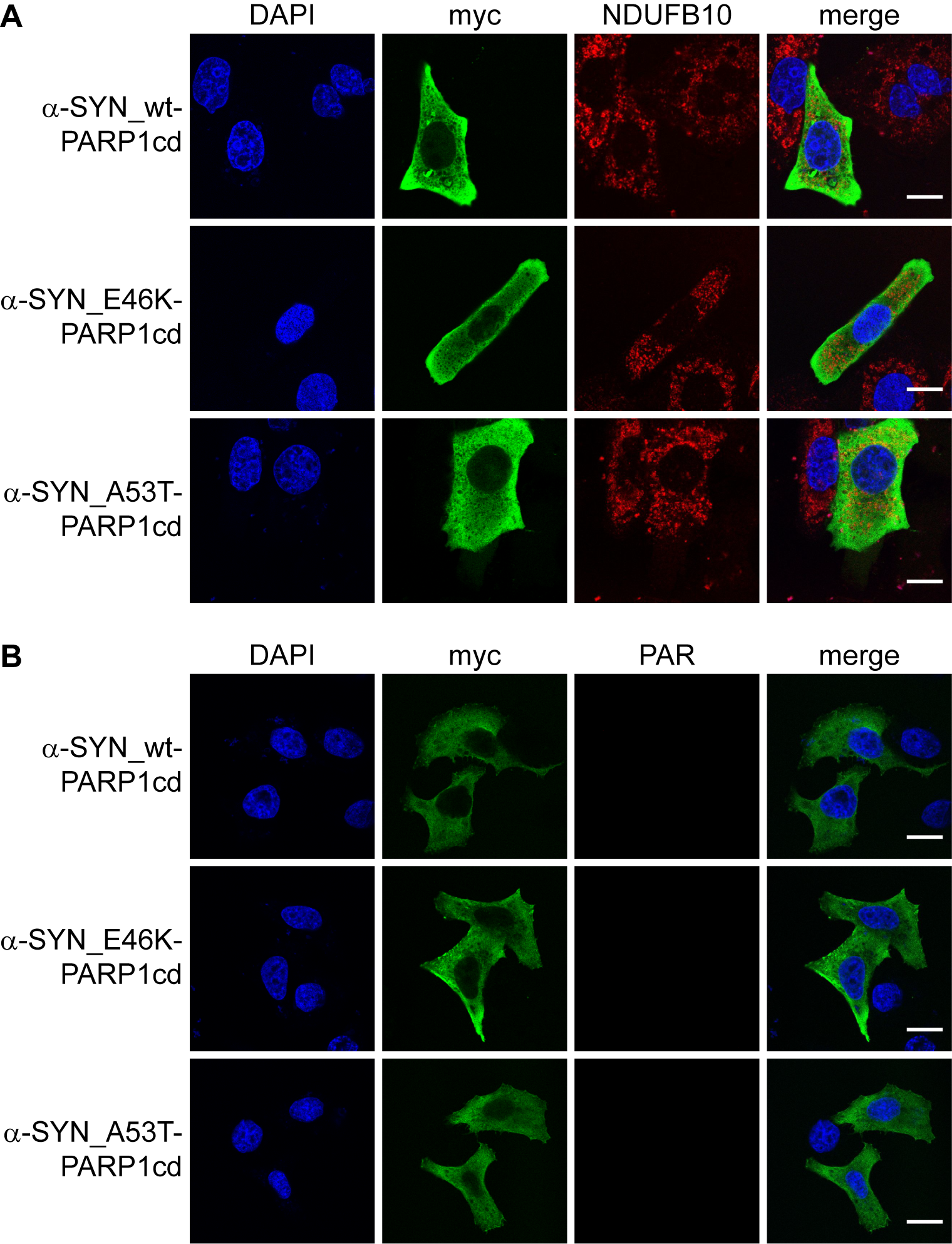

Supplement: S9 Fig — HeLa S3 cells, transiently transfected with PARP1cd fusion constructs of α-synuclein wt and mutants (E46K and A53T), were treated 24 hours after transfection with 20 μM CCCP for 6 hours and subsequently subjected to indirect immunocytochemistry, detecting the recombinant protein by its myc epitope and either a mitochondrial marker (A) or PAR accumulation (B). A) The fluorescent images show the overexpressed proteins (myc), mitochondria (NDUFB10) and nuclei (DAPI). Scale bar: 10 μm. (B) The fluorescent images show the overexpressed proteins (myc), PAR accumulation (PAR) and the nuclei (DAPI). Scale bar: 10 μm. (TIF) [file pone.0219909.s009.tif]

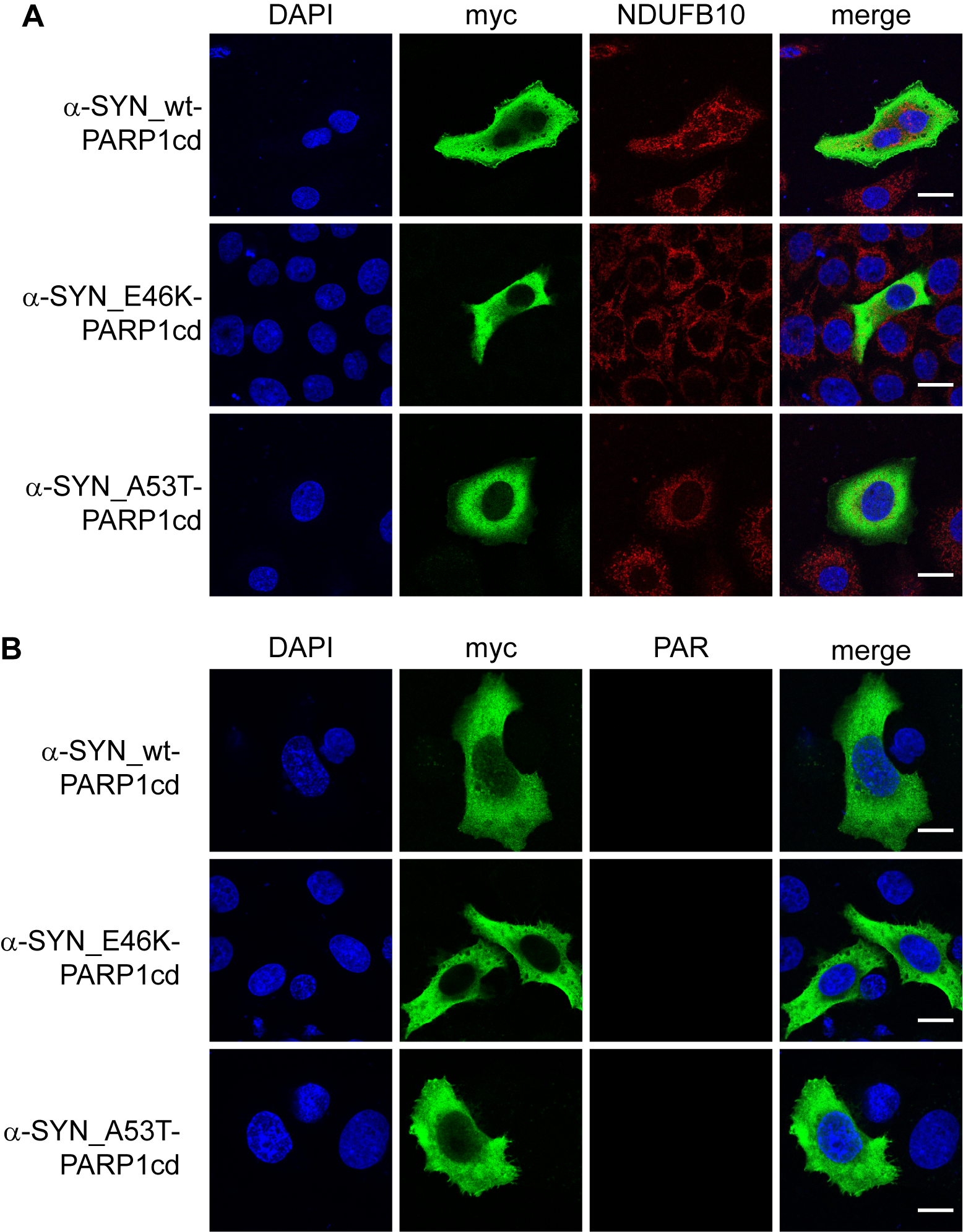

Supplement: S10 Fig — HeLa S3 cells, transiently transfected with PARP1cd fusion constructs of α-synuclein wt and mutants (E46K and A53T), were treated 24 hours after transfection with 2 mM paraquat for 24 hours and subsequently subjected to indirect immunocytochemistry detecting the recombinant protein by its myc epitope and either a mitochondrial marker (A) or PAR accumulation (B). (A) The fluorescent images show the overexpressed proteins (myc), mitochondria (NDUFB10) and nuclei (DAPI). Scale bar: 10 μm. (B) The fluorescent images show the overexpressed proteins (myc), PAR accumulation (PAR) and the nuclei (DAPI). Scale bar: 10 μm. (TIF) [file pone.0219909.s010.tif]

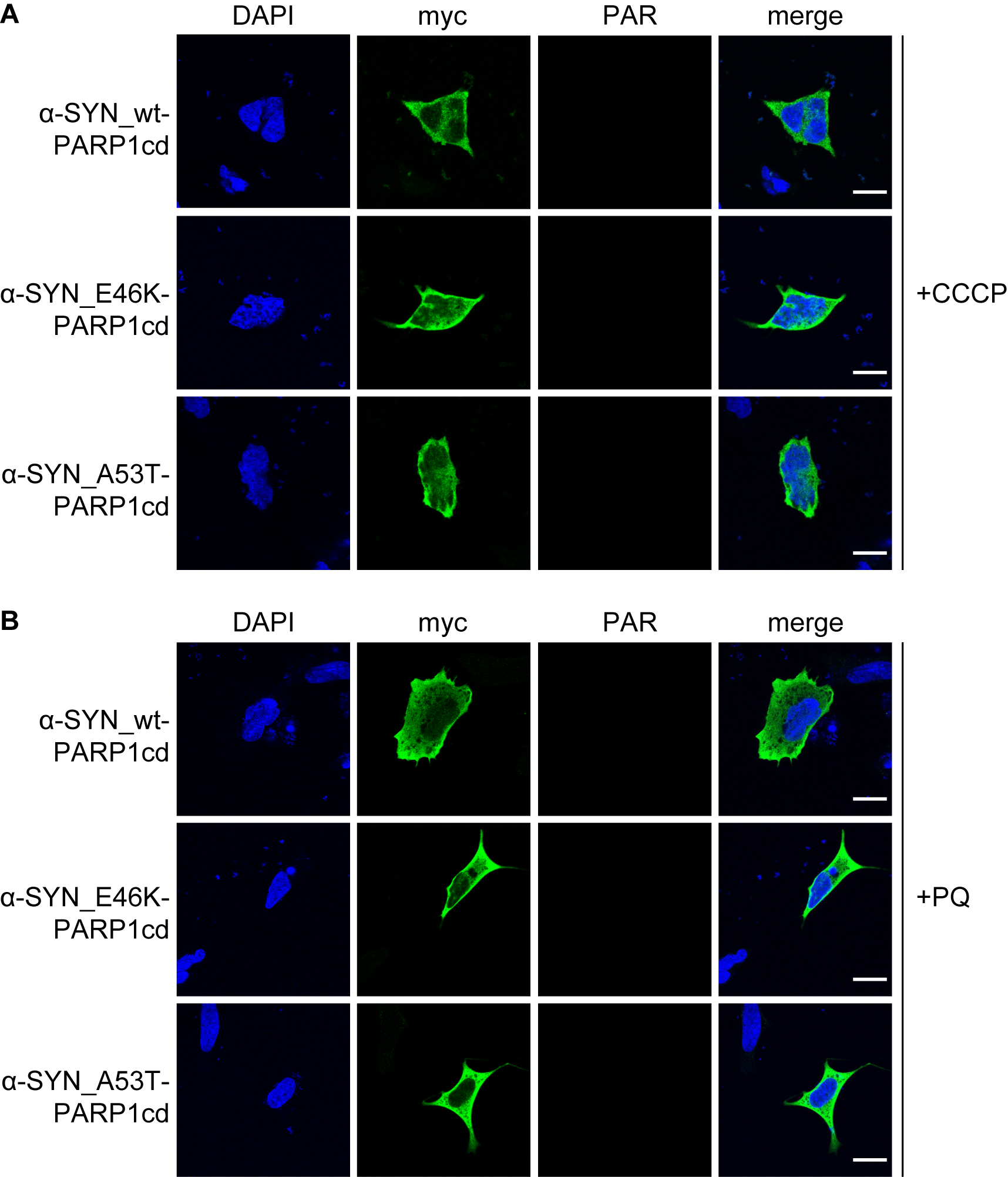

Supplement: S11 Fig — SH-SY5Y cells transiently transfected with PARP1cd-fusion constructs of α-synuclein wt and mutants (E46K and A53T), were treated 24 hours after transfection with 20 μM CCCP for 6 hours (A) or 1 mM paraquat (PQ) for 24 hours (B) and subsequently subjected to indirect immunocytochemistry detecting the recombinant protein and PAR accumulation. The fluorescent images show the overexpressed proteins (myc), PAR accumulation (PAR) and nuclei (DAPI). Scale bar: 10 μm. (TIF) [file pone.0219909.s011.tif]
